# Global transcriptomic analyses of *Salmonella enterica* in Iron-depleted and Iron-rich growth conditions

**DOI:** 10.1186/s12864-019-5768-0

**Published:** 2019-06-13

**Authors:** Bijay K. Khajanchi, Joshua Xu, Christopher J. Grim, Andrea R. Ottesen, Padmini Ramachandran, Steven L. Foley

**Affiliations:** 1 National Center for Toxicological Research, U. S. Food and Drug Administration, Jefferson, AR USA; 2 Center for Food Safety and Applied Nutrition, U. S. Food and Drug Administration, Laurel, MD USA; 3 Center for Food Safety and Applied Nutrition, U. S. Food and Drug Administration, College Park, MD USA

**Keywords:** *Salmonella*, Iron acquisition systems, Transcriptomic analysis, Iron-depleted growth conditions, Iron-rich growth conditions

## Abstract

**Background:**

*Salmonella enterica* possess several iron acquisition systems, encoded on the chromosome and plasmids. Recently, we demonstrated that incompatibility group (Inc) FIB plasmid-encoded iron acquisition systems (Sit and aerobactin) likely play an important role in persistence of *Salmonella* in human intestinal epithelial cells (Caco-2). In this study, we sought to determine global transcriptome analyses of *S. enterica* in iron-rich (IR) and iron-depleted (ID) growth conditions.

**Results:**

The number of differentially-expressed genes were substantially higher for recipient (SE819) (*n* = 966) and transconjugant (TC) (*n* = 945) compared to the wild type (WT) (SE163A) (*n* = 110) strain in ID as compared to IR growth conditions. Several virulence-associated factors including T3SS, flagellin, cold-shock protein (*cspE*), and regulatory genes were upregulated in TC in ID compared to IR conditions. Whereas, IS1 and *acrR*/*tetR* transposases located on the IncFIB plasmid, ferritin and several regulatory genes were downregulated in TC in ID conditions. Enterobactin transporter (*entS*), iron ABC transporter (*fepCD*), colicin transporter, IncFIB-encoded enolase, cyclic di-GMP regulator (*cdgR*) and other regulatory genes of the WT strain were upregulated in ID compared to IR conditions. Conversely, ferritin, ferrous iron transport protein A (*feoA*), IncFIB-encoded IS1 and *acrR*/*tetR* transposases and ArtA toxin of WT were downregulated in ID conditions. SDS-PAGE coupled with LC-MS/MS analyses revealed that siderophore receptor proteins such as chromosomally-encoded IroN and, IncFIB-encoded IutA were upregulated in WT and TC in ID growth conditions. Both chromosome and IncFIB plasmid-encoded SitA was overexpressed in WT, but not in TC or recipient in ID conditions. Increased expression of flagellin was detected in recipient and TC, but not in WT in ID conditions.

**Conclusion:**

Iron concentrations in growth media influenced differential gene expressions both at transcriptional and translational levels, including genes encoded on the IncFIB plasmid. Limited iron availability within the host may promote pathogenic *Salmonella* to differentially express subsets of genes encoded by chromosome and/or plasmids, facilitating establishment of successful infection.

**Electronic supplementary material:**

The online version of this article (10.1186/s12864-019-5768-0) contains supplementary material, which is available to authorized users.

## Background

*Salmonella enterica* is one of the major foodborne pathogens in the United States [1, 2], often associated with multistate outbreaks linked to contaminated foods and food products or pet animals such as turtle [3] and hedgehogs [4]. *Salmonella* can cause a wide range of human infections, from mild gastroenteritis to invasive diseases [5]. Over 2600 *Salmonella* serovars have been identified, largely differing in host ranges and their ability to cause human infections [5]. *Salmonella enterica* serovars such as Enteritidis, Typhimurium, Newport, and Heidelberg can colonize intestines of a broad range of hosts including food producing animals and humans [6]. On the other hand, some *Salmonella* serovars are host-restricted such as Typhi, Paratyphi, Gallinarum, Choleraesuis, Abortusovis and Dublin, which can only cause infections in one or few hosts [7]. Nonetheless, genetic factors that contribute to boarder host range and increased ability to cause invasive form of disease to different *Salmonella* serovars are still largely unknown.

*Salmonella* possess arrays of genes that aid in invasion, replication, and persistence inside the host cells [8]. Type III secretion systems (T3SS) are among the major factors that play roles in invasion and persistence in the host cells [9–11]. *Salmonella* pathogenicity islands (SPIs) encode virulence factors, including T3SS, that are required during infections of host cells [12]. Genomes of *Salmonella* acquire SPIs through different evolutionary processes via horizontal gene transfer (HGT). To date, 21 SPIs have been identified in *S*. Typhimurium, among which SPI-1 and SPI-2 are well studied [9–11, 13]. SPI-1 contains genes, which are essential for the early infection process to host cells, while SPI-2 contains genes that contribute to late infection processes of survival and replication inside host cells [14]. SPI-1 and SPI-2 harbor T3SS-1 and T3SS-2, respectively, and regulate expression of virulence genes including secretion of T3SS effector proteins in a highly regulated manner to establish infection in the host [15–17]. Besides chromosomally-mediated virulence factors, we and others have demonstrated that virulence plasmid-encoded factors such as iron acquisition systems located on incompatibility group (Inc) FIB plasmids [18], VirB/D4 type 4 secretion systems encoded on the IncX4 plasmid, [19] and the *spv* operon located on multiple virulence plasmids [20] likely contribute to increased virulence of *Salmonella* during infection of host cells. However, the precise role of these virulence-associated plasmid factors of *Salmonella* in infection process remains to be determined.

*Salmonella* serovars encounter different challenging environments during host-pathogen interactions, including iron-limited conditions inside the host cells. Iron is not only an essential growth factor for many pathogenic bacteria [21], but also serves as a signaling element that regulates various genes, including virulence associated genes [22]. The master regulator, Fur (ferric uptake regulator), senses iron availability and controls gene expression as necessary, in response to physiological conditions [23–25]. Previous studies have shown that a Fur mutant attenuated the virulence and pathogenesis observed in vivo models for several pathogens [24, 26–29].

For most bacteria, iron acquisition is one of the key factors that determine their ability to survive in a host [30]. Bacteria that do not possess iron acquisition capability, may be removed by the host defense mechanisms [30]. Some of the common host defense mechanisms are: i) to limit iron availability to pathogen by forming iron-host protein complexes. The host produces a specific class of the glycoprotein transferrin, such as serotransferrin and lactoferrin, which have iron-binding capability [30–32]. ii) to respond directly to invasive pathogens by altering the status of internal host iron concentrations. Host cells, such as macrophages produce Nramp (natural resistance-associated macrophage protein) proteins that control internal iron status in response to infection [30]; and iii) to impact host defense cells, such as macrophages which require iron for production of toxic reactive oxygen intermediates (ROI) and reactive nitrogen intermediate (RNI) to stimulate oxidative damage to eliminate pathogens [21].

*Salmonella* have evolved with different iron acquisition systems, encoded on the chromosome and plasmids, which facilitate them to chelate iron from the host to establish successful infection [22, 33]. A previous study showed that the *sitABCD* iron transporter operon located on the SPI-1, is induced under iron-depleted conditions [34]. Additionally, they demonstrated that genes of the *sitABCD* operon are repressed by Fur [34]. The same study showed that the SitABCD (Sit) system was increasingly expressed after invasion of the intestinal epithelium and a Sit null mutant was attenuated in animal experiment [34]. These data indicate that chromosome-encoded Sit iron transport system contributed to virulence of *S*. Typhimurium. Incompatibility (Inc) group FIB plasmids can also encode the Sit and the aerobactin iron acquisition (*iucABCD-iutA*) systems [18, 35]. Mechanisms of regulation of plasmid-mediated Sit and aerobactin iron acquisition systems have yet to be determined.

*Salmonella* possess several regulatory systems that facilitate control of gene expression in a highly regulated manner to survive in different environmental niches [36–38]. A previous study investigated transcriptomic analyses of 22 distinct infection-relevant environment conditions to mimic infection in host using RNA-Seq [38]. They showed that different in vitro conditions stimulate characteristic transcriptional signatures genes, in which 22 conditions influenced transcriptions of 86% of total genes of *S*. Typhimurium [38]. Additionally, Hautefort et al. (2008) demonstrated that *S*. Typhimurium undergoes time-dependent transcriptional adaptation resulting simultaneous expression of three T3SS systems during infection of epithelial cells [39].

In a recent study, we demonstrated that IncFIB plasmid-encoded iron acquisition systems (Sit and aerobactin) likely play an important role in persistence of *Salmonella* in human intestinal epithelial cells [18]. In this study, we sought to determine the global iron regulated transcriptomes and proteome levels of selected protein bands of *S. enterica*. We identified differential expression of several genes located in the chromosome and on IncFIB plasmids at the transcriptional level using RNA-Seq. Selected differentially-expressed proteins were also determined by nano-liquid chromatography-tandem mass spectrometry (LC-MS/MS). The finding of the study will be useful for better understanding the influence of iron on the transcriptomes and proteomes of *Salmonella.*

## Methods

### Bacterial strains and growth medium

*Salmonella enterica* recipient SE819 and wild type SE163A strains isolated from food sources were sequenced in our laboratory [40]. A transconjugant (SE819::IncFIB) was developed in our previous study [18]. Ferric chloride (100 μM) and 2,2^/^−bipyridyl (200 μM) (Sigma-Aldrich, St. Louis, MO) were added to LB broth to prepare iron-rich (IR) and iron-depleted (ID) growth media, respectively as described in previous studies [41, 42].

### RNA isolation and quality assessments

Bacterial strains were sub-cultured on sheep’s blood agar plates (Remel, Lenexa, KS) and single colonies of wild type, recipient, and transconjugant (SE819::IncFIB) strains were picked and grown in IR and ID LB broth overnight at 37 °C with shaking. The next day, overnight cultures were diluted at ratio of 1:20 in to corresponding fresh IR and ID LB medium. RNA was isolated from approximately 10^9^ cells collected at mid-logarithmic phase from two biological replicates of each strain with the Ribopure Bacterial RNA Isolation Kit (Ambion, Invitrogen, Carlsbad, CA), following manufacturer’s instructions. Genomic DNA was removed by treatment with DNase I (Ambion, Invitrogen, Carlsbad, CA).

Total RNA concentration was measured with Qubit fluorometer using RNA BR Assay kit (ThermoFisher Scientific, Waltham, MA), and the quality of RNA was examined by determining RNA integrity number (RIN) using Agilent Bioanalyzer 2100 with RNA 6000 nano kit (Agilent, Santa Clara, CA). Samples with RIN values ≥7 were subjected to library preparation for RNA-seq.

### Library preparation and RNA sequencing

rRNA was depleted from 5 μg of total RNA from each sample using the ribo-zero rRNA removal kit (Illumina, San Diego, CA) according to instruction manual. Briefly, for each reaction, in the first step 225 μL magnetic beads were washed. Then, ribo-zero removal solution was added to each RNA sample in order to hybridized probe to rRNA present in the samples. Washed beads and probe-hybridized samples were mixed together to remove rRNA from the samples. rRNA depleted samples were cleaned up by standard ethanol precipitation, and the quality of rRNA-depleted samples was assessed using the Agilent Bioanalyzer 2100 as described above.

RNA-Seq libraries were prepared by Truseq Stranded mRNA kit (Illumina, San Diego, CA) using 160 ng of each rRNA depleted sample. Briefly, first and second strand cDNA was synthesized and adenylated 3^/^ ends of the blunt fragments to prevent them from ligating to one another during the adaptor ligation step. After that adaptors were ligated and selective DNA fragments with adaptors were enriched using PCR performed with a PCR primer cocktail that anneals to the ends of the adapters. The quality of cDNA was examined using Bioanalyzer with Agilent DNA 1000 kit (Agilent, Santa Clara, CA). After validation, libraries were quantified using KAPA NGS library quantification kit (Kapa Biosystem, Wilmington, MA) by quantitative reverse transcription-PCR (qRT-PCR) using SYBR green assays (Bio-Rad, Hercules, CA). RNA-Seq libraries were normalized, pooled, and sequenced by Illumina NextSeq platform using 2 × 75 pair-end (PE) format. RNA sequencing was performed at Center for Food Safety and Applied Nutrition, FDA, College Park, MD.

FASTQ files generated from NextSeq were imported into CLC Genomics Work Bench version 9.0 (Qiagen, Redwood City, CA). In order to accommodate genes located on the IncFIB plasmid, sequence reads were mapped to the genome sequences of *S.* Typhimuirum LT2 (lacks IncFIB plasmid) and the WT SE163A. For transcriptome analyses, a combined gene track from annotated draft genome of SE163A and the complete genome of LT2 were established using CLC Genomics Workbench. The gene name indicated by AY603_XXXXX is mapped from SE 163A and the rest of the genes are mapped from LT2. Transcripts per million (TPM) was calculated for individual genes using CLC Genomics Workbench. Comparison of differential gene expression between two groups was conducted and fold-changes were determined by ‘Exact Test’ [43] and EdgeR [44], a bioinformatic tool for empirical analysis of differential gene expressions (designated as experimental fold-changes). Additionally, TPM of each gene was normalized with geometric mean TPM value of *gmk* and *adk* and fold-changes were calculated from normalized values (designated as normalized fold-changes). Normalized value with ≥4-fold differences of transcript abundances between ID and IR growth conditions were considered to demonstrate the presence of differential changes in gene expression (upregulation or downregulation) among the *Salmonella* strains compared. Total of 12 RNA sample libraries, representing two biological replicates for each strain in two different conditions (ID and IR) were included in the RNA-seq experiment.

### Quantitative RT-PCR

qRT-PCR using SYBR green assays [18] was performed to validate gene expression levels obtained in RNA-Seq. qRT-PCR on selected genes such as *entS*, *fepC*, enolase, and colicin transporter (colicin-T) was performed using same RNA sample preparation that were used in RNA-Seq, but with different newly generated cDNA. The primers were designed using the PrimeQuest tool (Integrated DNA Technologies, Coralville, IA) (Additional file 5: Table S5) and the cDNA was synthesized from 250 ng of RNA using the iScript cDNA synthesis kit (Bio-Rad, Hercules, CA). qRT-PCR was performed on CFX touch real-time PCR detection system (Bio-Rad, Hercules, CA) and the *gmk* and *adk* genes of *Salmonella enterica* were used as endogenous control to normalize expression values. Differential gene expression and fold changes were determined using Bio-Rad CFX manager software.

### Proteome analysis of select protein bands

Wild type, recipient, and transconjugant strains were grown in IR and ID LB broth overnight at 37 °C with shaking. Overnight cultures were diluted (1:20) in to corresponding fresh IR and ID LB media and cell lysates were prepared at mid-logarithmic phase. To determine the approximate bacterial cell numbers, optical density (OD) was measured at 600 nm, using a Genesys 10UV spectrophotometer (Thermo Electron Corp., Madison, WI). Bacterial cell pellets were dissolved in protein sample buffer (Bio-Rad, Hercules, CA), boiled at 100 °C for 10 min and 25 μL of cell lysates containing equal number of bacterial cells (~ 10^8^ cells) were loaded onto a 4 to 15% gradient Criterion TGX precast protein gel (Bio-Rad, Hercules, CA). Protein bands were separated on a Criterion electrophoresis chamber and visualized by staining with Coomassie blue. Triplicate protein gels were run and stained using protein lysates collected from three independent experiments in order to examine the consistent differential expressions of protein bands from each experiment. Differently expressed protein bands were selected from one representative gel, cut and sent to MS Bioworks (Ann Arbor, MI) for further proteome analyses.

At MS Bioworks, protein content in gel bands were identified by nano-liquid chromatography-tandem mass spectrometry (LC-MS/MS) as described earlier [45].

## Results

**Fig. 1:**
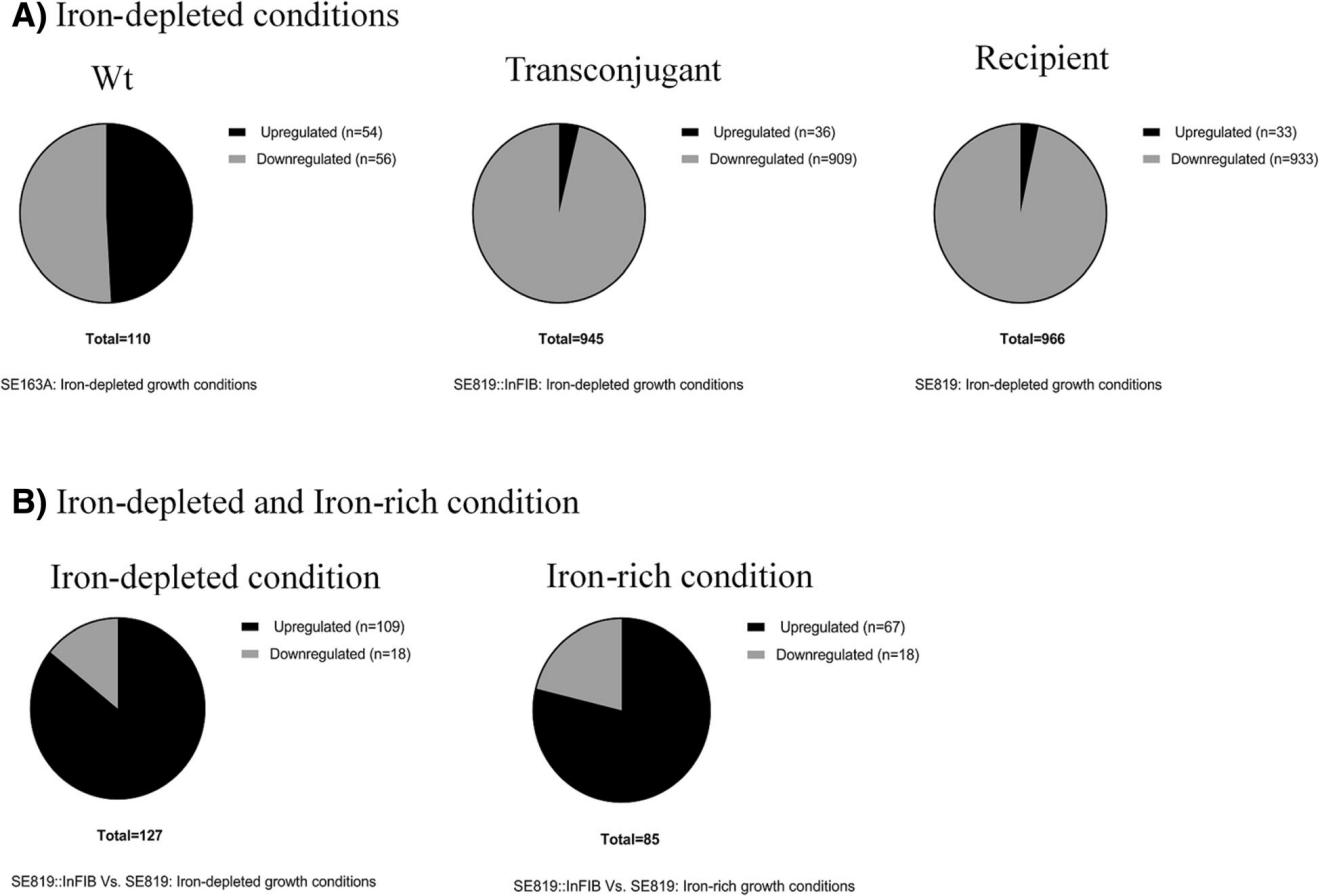
Global transcriptomic analyses of *S. enterica* by RNA-Seq. Total of 12 RNA sample libraries, two biological replicates for each strain in two different conditions (ID versus IR) were included in the RNA-Seq experiment. Sequence reads were mapped to genome sequences of *S.* Typhimuirum LT2 and the WT SE163A. Transcripts per million (TPM) was calculated for individual genes. TPM of each gene was normalized with *gmk* and *adk* and fold-changes were calculated from normalized values. A cut off ≥4-fold differences of transcript abundances was used to establish the presence of differential gene expression. **a**) Less number of genes were differentially expressed in WT as compared to transconjugant (SE819::IncFIB) and recipient (SE819) strains in iron-depleted conditions. **b**) More number of genes were up-regulated in ID as compared to IR growth conditions in transconjugant than recipient. Whereas, identical number of genes were downregulated in these two strains in IR and ID growth conditions

To determine influence of iron on the transcriptomes of WT (SE163A), transconjugant (TC) (SE819::IncFIB) and recipient (SE819) *Salmonella enterica* strains, RNA-Seq analyses were performed using RNA extracted from these strains in IR and ID growth conditions. A goal of the study was to determine the differential gene expression of chromosomally- and plasmid-encoded genes including IncFIB plasmids. Hence, we mapped the sequence reads with the genome sequence of SE163A and *S*. Typhimurium LT2 to accommodate the genes present in WT, TC and recipient strains. The number of differentially expressed genes were substantially higher for recipient (*n* = 966) and TC (*n* = 945) compared to the WT (*n* = 110) strain in ID as compared to IR growth conditions (Fig. 1a). Fifty-four genes were upregulated in ID as compared to IR for the WT strain. These numbers were 36 and 33 for TC and recipient, respectively. Whereas, 56, 909, and 933 genes were downregulated in ID as compared to IR in WT, TC and recipient strains, respectively (Fig. 1a). In ID growth conditions, 109 genes were upregulated whereas 18 genes were downregulated in TC as compared to recipient, while, these numbers were 67 and 18 in IR growth conditions, respectively (Fig. 1b). Gene ID, potential functions and normalized fold changes of differentially expressed genes of WT and TC in ID as compared to IR growth conditions are listed in supplemental document Additional file 1: Table S1, Additional file 2: Table S2, Additional file 3: Table S3 and Additional file 4: Table S4.

Selected genes with higher fold changes that were differentially expressed (upregulated or downregulated) in WT and TC were listed in the Tables 1, 2, 3 and 4. Several virulence associated factors including T3SS, flagellin, cold-shock protein (*cspE*), and other regulatory genes were upregulated in TC in ID compared to IR conditions (Table 1). Whereas, IS1 and *acrR/tetR* transposases, ferritin, sensor protein (*basS/pmrB*), nucleoside triphosphatase (*nudI*), zinc transporter (*zupT*) and several regulatory genes were downregulated in TC in ID conditions (Table 2). For the WT strain, alkylhydroperoxidase (*yciW*), enterobactin transporter (*entS*), iron ABC transporter (*fepCD*), colicin transporter, IncFIB encoded enolase, cyclic di-GMP regulator (*cdgR*) and other regulatory genes were upregulated in ID compared to IR conditions (Table 3). Like TC, IS1 and *acrR/tetR* transposases, ferritin, sensor protein (*basS/pmrB*), zinc transporter (*zupT*), and nucleoside triphosphatase (*nudI*) were also downregulated in ID in WT along with other genes including ferrous iron transport protein A (*feoA*), and IncFIB encoded toxin gene *artA* (Table 4).

**Table 1:**
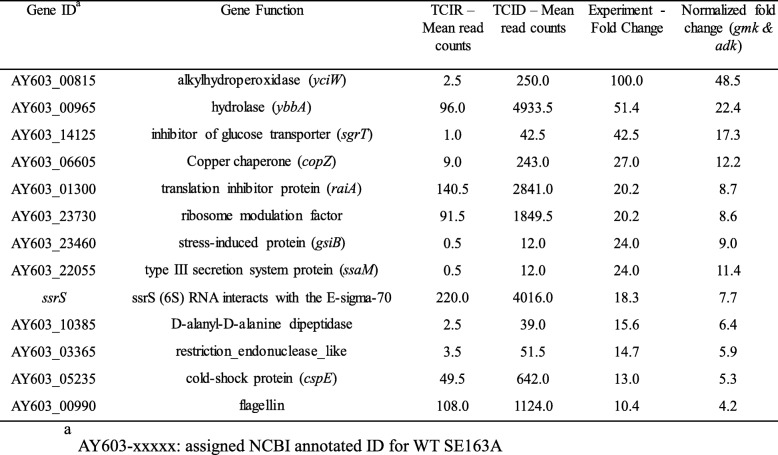
Selected genes that were upregulated in transconjugant SE819::IncFIB in iron-depleted as compare to iron-rich conditions in RNA-Seq

**Table 2:**
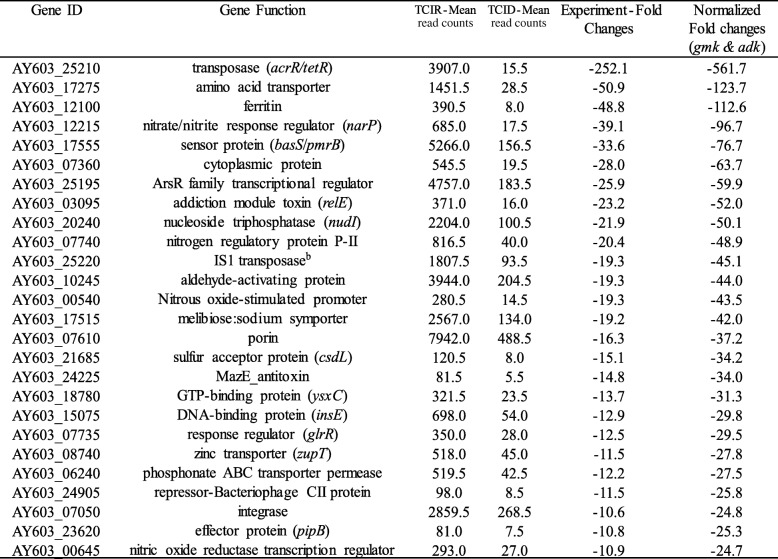
Selected genes that were downregulated in transconjugant SE819::IncFIB in iron-depleted as compare to iron-rich conditions in RNA-Seq

**Table 3:**
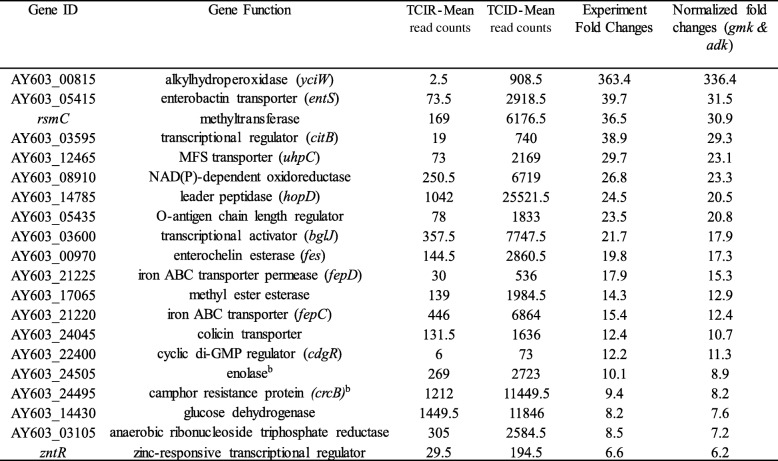
Selected genes that were upregulated in WT SE163A in iron-depleted conditions as compare to iron-rich in RNA-Seq

**Table 4:**
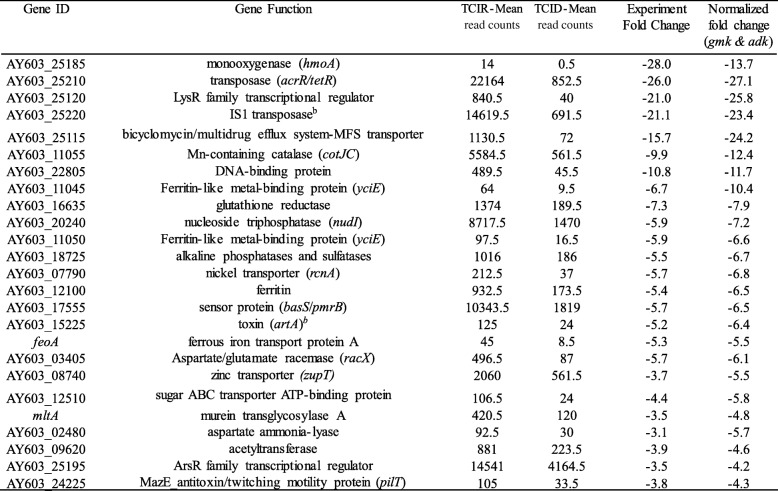
Selected genes that were downregulated in WT SE163A in iron-depleted as compare to iron-rich conditions in RNA-Seq

**Fig. 2:**
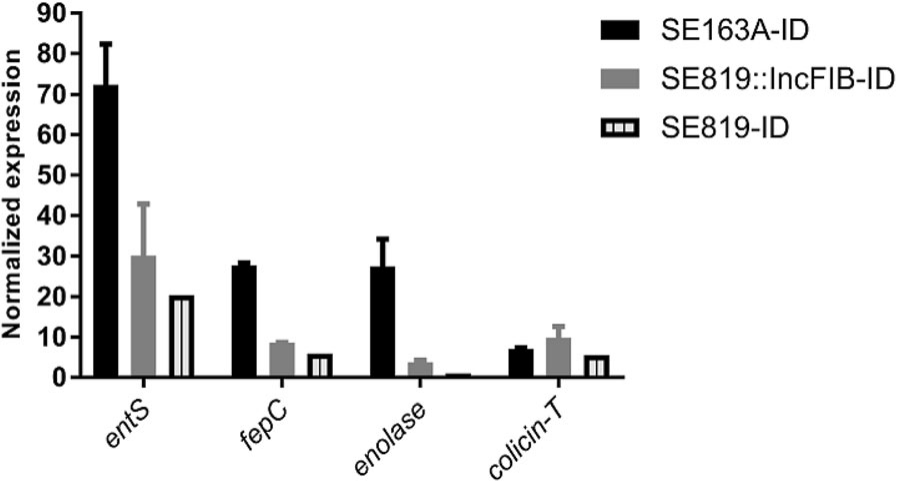
Validation of RNA-Seq data by qRT-PCR. Quantitative reverse transcription-PCR (qRT-PCR) using SYBR green assays was performed to validate gene expression levels obtained by RNA-Seq. Four genes [*entS*, *fepC*, enolase, colicin transporter (*colicin-T*)] were selected to validate the RNA-Seq results of WT, transconjugant and recipient. Two biological replicates and three technical replicates were used for each sample of two different conditions (ID and IR). Gene expression was normalized by *gmk* and *adk* reference genes. In the figure, each bar indicates the increased fold changes of that gene corresponding to the same strain in ID as compared to IR conditions. For example, in WT (SE163A) *entS* transcript was increased approximately 70-fold in ID as compared to IR. Error bars indicate standard error of mean (SEM) of gene expression of two biological replicates for each strain

Transcriptomic results obtained from RNA-Seq analyses were validated by qRT-PCR. Transcription of four genes (*entS*, *fepC*, *enolase*, colicin transporter) in WT, transconjugant and recipient (Fig. 2) were examined by qRT-PCR using same RNA preparation that were used for RNA-Seq. All four genes were upregulated with highest fold changes (70-fold) observed for *entS* in WT strain in ID as compared to IR conditions, these data match with RNA-Seq analyses (Fig. 2). These genes were also upregulated in TC and recipient in ID albeit at lower fold-change values as compared to the WT (Fig. 2). Both RNA-Seq and qRT-PCR data demonstrated that the enolase gene located on the IncFIB plasmid was upregulated in the WT strain as compared to TC in ID growth conditions.

**Fig. 3:**
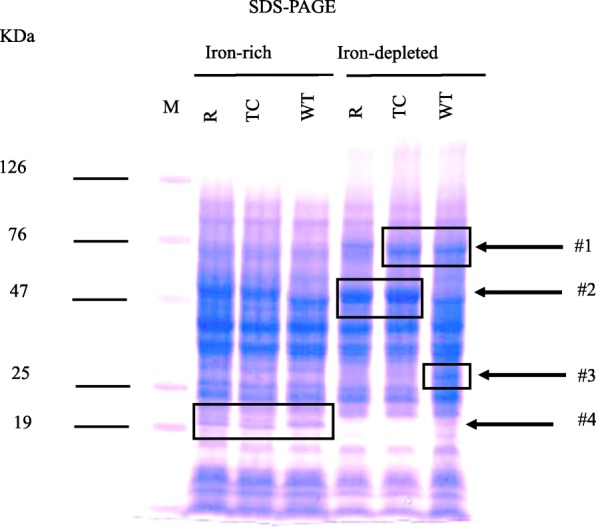
Protein bands were separated on SDS-PAGE gel for proteome analysis. Proteome analyses of WT, transconjugant and recipient were performed after grown in IR and ID conditions using SDS-PAGE and selected protein bands were identified by LC-MS/MS analyses as indicated in the figures (Bands positions 1 to 4). SDS-PAGE analyses showed that Band #1 was increasingly expressed in TC and WT as compared to recipient. Whereas, Band #2 was increasingly expressed in TC and recipient as compared to WT. Band #3 was only expressed in WT not in TC and recipient. Band # 4 was increasingly expressed in WT, TC and recipient in IR as compared to ID conditions. M = protein ladder; R = recipient; TC = transconjugant; WT = wild type

To examine if iron also influences expression of proteins, proteome analyses of WT, transconjugant and recipient were performed in IR and ID growth conditions using SDS-PAGE (Fig. 3) and selected differentially expressed proteins in these two conditions were identified by LC-MS/MS analyses (Table 5). Differential protein expressions were observed in these strains in ID as compared to IR as examined by SDS-PAGE (Fig. 3). Our data showed that several proteins were upregulated in ID as compared to IR conditions. Band #1 in SDS-PAGE was upregulated in TC and WT as compared to recipient in ID growth conditions. These protein bands (Band #1), identified as siderophore receptors, IroN and IutA, are located on the chromosome and the IncFIB plasmid, respectively (Table 5). Band #2 in SDS-PAGE was upregulated in TC and recipient as compared to WT (Fig. 3) and was identified as flagellin, FljB (Table 5). Band #3 was only expressed in WT and not in TC and recipient, and was identified as iron acquisition protein, SitA, which is located on the chromosome as well as on the IncFIB plasmid. Lastly, Band # 4 was overexpressed in these three strains in IR as compared to ID conditions. Band #4 was detected as a ribosomal protein L5, RplE.

**Table 5:**
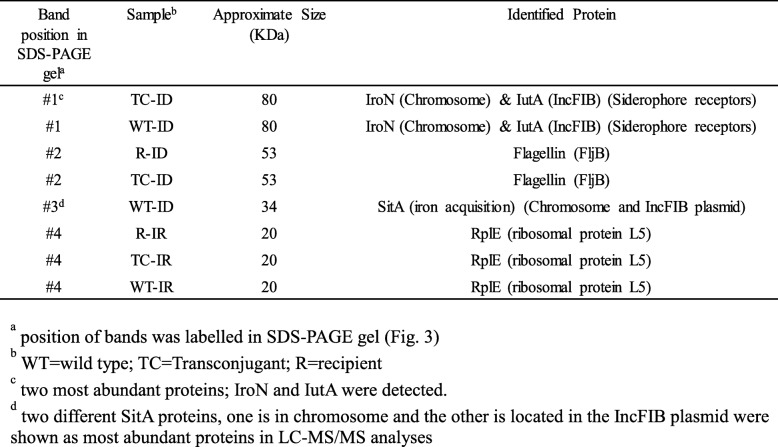
Selected protein bands of WT, transconjugant and recipient of *Salmonella enterica* were identified by LC-MS/MS

## Discussion

Iron is an important signaling element that regulates a vast array of genes including virulence factors, as well as an essential growth factor, for many bacterial pathogens to achieve virulence during infection [46–51]. Since bacteria encounter iron limited conditions within the host, bacteria must chelate iron to overcome host sequestration in order to survive and establish infection [21, 51]. Several iron acquisition systems including Sit, aerobactin (Iuc) and Feo system for ferrous iron utilization [34, 52] have been identified in bacteria and located in the chromosome and/or on plasmids. For example, Sit and Iuc have been found to be located on IncFIB plasmids [33, 35]. A previous study demonstrated that deletion of all three iron acquisition systems, namely Sit, Iuc and Feo, abolished the ability of *Shigella flexneri* to form plaque on tissue culture monolayers [53]. The same study showed that a single knock-out of the Sit system resulted in a mutant strain that retained its ability to form plaque similar to WT level while double knockout mutants in different combination such as Sit/IuC or Sit/Feo or Feo/IuC formed smaller plaques as compared to the WT [53]. Other studies have also shown that deletion of the Sit system, located on the chromosome, resulted in a decreased ability of *S*. *flexneri* as well as *S*. Typhimurium to cause infection in an animal host [34, 52]. The findings of these studies revealed that all the three iron acquisition systems likely contribute to virulence of *S*. *flexneri* and related pathogens. We and other investigators demonstrated that IncFIB encoded iron acquisition systems (Sit and aerobactin) likely contribute to increased ability to colonize chickens [54] and increased persistence in human epithelial cells (Caco-2) [18]. Nonetheless, the roles of iron acquisition systems, located on plasmids, in virulence and the mechanisms of how iron regulates iron acquisition genes residing on plasmids of *Salmonella*, has yet to be determined. In the present study, we demonstrated that iron influenced expression of a number of genes located on both chromosome and on the IncFIB plasmid of *S. enterica*, both at the transcriptional and translational levels.

In our previous study we demonstrated that the transconjugant (SE819::IncFIB) persisted in Caco-2 cells at a significantly higher rate than the recipient SE819 after 48 h of post infection when these strains were grown in standard LB or iron-depleted LB, but not in iron-rich LB prior to infection [18]. Additionally, we noticed that WT strain, SE163A, was able to invade and persist in Caco-2 cells at much higher rate than the recipient and TC of *S. enterica* [18]. To determine how iron levels, influence the gene expression profiles of these three *S. enterica* strains (WT, TC and recipient), we performed transcriptomic analysis in ID and IR growth conditions using RNA-seq. In order to reduce the background gene expression variability among the strains, a cutoff point ≥4 fold-change differences except for *fepB* and another transporter gene with ≥3 fold changes (Additional file 3: Table S3), was employed to determine differential gene expression between strains and iron growth conditions. In addition, fold-changes were determined in two different ways; i) experiment fold-changes were determined by exact test using EdgeR by CLC genomic workbench; and ii) fold-changes of gene expression obtained in RNA-Seq were normalized by the gene expression of two housekeeping genes, *gmk* and *adk*. Previous studies confirmed that expression of these genes was stable in different conditions tested, and hence suitable for use as reference genes for normalizations for both RNA-Seq and qRT-PCR analyses [55, 56]. Although we listed both the experiment fold-change and normalized fold-change in Tables 1, 2, 3 and 4; we identified the total number of genes that were differentially expressed between two groups of strains compared based on the normalized fold changes, as shown in Fig. 1a and b.

RNA-Seq analyses revealed that a higher number of genes were differentially expressed in transconjugant and recipient as compared to the WT strain in ID growth conditions (Fig. 1a and b). This distinct gene expression is likely due to different genetic background of these three-strains studied. WT strain may possess different genetic factors that contribute to increased ability to tightly regulate genes in iron limited conditions in order to deal with iron stress. These characteristics may contribute to increased virulence to WT strain as compared to TC and recipient strains. However, further experimental evidence is needed to substantiate our hypothesis.

More importantly, several genes were differentially expressed in ID and IR conditions in transconjugant as compared to the recipient strain (Fig. 1b). These data indicate that IncFIB encoded factors including iron acquisition systems may influence the gene expression of transconjugant in both ID and IR conditions. Several virulence associated genes including a T3SS factor (*ssaM*) and cold-shock protein (*cspE*) were upregulated in TC in ID as compared to IR (Table 1). A previous study demonstrated that low pathogenicity strains of *S*. Enteritidis showed distinct transcriptomes with significantly reduced expression of virulence and stress-associated genes compared to the high pathogenicity strains [57].

SPI-2 harbor multiple operons including T3SS-2 genes [58, 59]. T3SS-2 genes facilitate *Salmonella* to survive inside the host cells such as macrophage [60]. In the present study, we observed increased expressions of T3SS-2 genes such as *ssaM* and *sseA* (Table 1 and Additional file 1: Table S1) in ID compared to IR growth conditions in the transconjugant (SE819::IncFIB). Our data supported previous findings reported by Zaharik et al [61] where they demonstrated that SPI-2 genes were upregulated in ID growth conditions as compared IR condition. From these data, we speculate that increased expression of T3SS-2 genes may contribute to increased survival of the transconjugant bacteria in the host cells. However, additional studies are required to substantiate this speculation.

Our data showed that glucose transporter gene *ptsG* (AY603_16045), a component of phosphotransferase transferase system, was upregulated in WT in ID as compared to IR growth conditions (Additional file 3: Table S3). Several studies demonstrated that *ptsG* is a regulatory gene that control expression of various virulence associated genes that are often required for the establishment of infection in host [62, 63]. On the other hand, the small regulatory RNA SgrS (AY603_14125) that is an inhibitor of glucose transporter, (and encodes a small protein SgrT) is upregulated in ID compared to IR in transconjugant (Table 1). A recent study demonstrated that SgrS can perform dual functions, including controlling gene expressions post-transcriptionally via sRNA-mRNA base-pairing interactions and encoding a small protein named SgrT, which can inhibit the transport activity of PtsG [64]. Another small regulatory RNA (sRNA), SsrS (6S RNA) is upregulated in the transconjugant during ID growth conditions (Table 1). This result in agreement with findings in a previous study, where it was shown that 6S RNA in *Burkholderia cenocepacia* upregulated under iron depleted conditions [65]. Another study demonstrated that 6S RNA, encoded by SsrS, plays role in *S*. Typhimurium survival under acid stress [66]. Additionally, the same study demonstrated that a *S*. Typhimurium 6S RNA knockout strain showed reduced invasion ability in HeLa cells and decreased virulence in mouse model [66]. Nonetheless, the mechanisms of how iron limited conditions induce the expressions of sRNAs in WT and transconjugant stains in ID growth conditions in the present study remains to be determined. In the present study, increased expression of the virulence factors in ID conditions in TC but not in recipient may translate to increased virulence in iron limited conditions in the host during infection; however, the mechanisms of how iron depleted conditions influence regulation of these virulence genes in the TC but not in the recipient, is unknown.

Interestingly, IS1 and *acrR*/*tetR* transposes encoded on IncFIB plasmid, and chromosome encoded genes including ferritin, *basS*/*pmrB*, *nudI*, *zupT*, were downregulated in ID conditions both in TC and WT strains (Tables 2 and 4). However, enolase (upregulated) and *artA* (downregulated) genes encoded by the IncFIB plasmid were only differentially expressed in WT but not in TC in ID growth conditions suggesting that IncFIB plasmid encoded genes are likely distinctly regulated in WT (SE163A) as compared to the TC strain background tested ID and IR growth conditions.

A plasmid encoded colicin transporter gene (AY603_24045) was upregulated ~ 11 fold in ID compared to IR conditions in WT strain (Table 3). This result is in accordance to a previous finding of Spriewald et al. (2015), where they demonstrated that the ColIb gene (*cib*), was increasingly expressed in iron depleted conditions [67]. A previous study in our laboratory examined the distribution of *cib* in different *Salmonella* serovars isolated from poultry and food producing animals [68]. Our data showed that 35% *Salmonella* isolates (*n* = 32) were positive for *cib* among 92 isolates examined [68]. Ability of colicin production may provide competitive colonization advantage to pathogen including *Salmonella* [69, 70].

Similar to transcriptome analyses, our data showed that the three strains had distinct protein expression profiles as evidenced by proteome analyses of the selected protein bands using SDS-PAGE and LC-MS/MS (Fig. 3). Interestingly, siderophore receptors IroN (product of *iroN* located on the chromosome) and IutA (product of *iutA* located on the IncFIB plasmid) proteins were increasingly expressed in WT and TC in ID conditions as compared to recipient, albeit the recipient lacks the IncFIB plasmid (Fig. 3). Conversely, SitA protein (product of *sitA* gene, located on chromosome as well as on IncFIB plasmid) was expressed only in WT in ID conditions and not in TC and recipient. Sequence analyses showed that the *sitA* located on the chromosome is different than the *sitA* gene located on the IncFIB plasmid [18]. Increased expression of SitA protein located on the IncFIB plasmid in ID conditions may reflect that plasmid-encoded SitA likely plays a role in iron depleted conditions. Nonetheless, the biological functions of plasmid encoded iron acquisition systems, including Sit, are currently unknown. In future studies, we plan to examine the specific functions of plasmid encoded iron acquisition systems in the virulence of *S. enterica*. The proteomic analyses performed on the selective set of protein bands, showed differential expression of proteins (IroN, IutA, FljB, SitA) in WT, transconjugant and recipient were distinct from the expression of these genes at the transcription level observed by RNA-Seq analyses. The reasons for the distinct expression profiles remains unknown and may be explored further in future efforts.

## Conclusion

Overall, the present study provides important findings as to how iron influences different genes of *S. enterica* at the transcriptional and translational levels in isolates that carry iron acquisition systems encoded both on the chromosome and on plasmids such as IncFIB in this study (WT and TC). Based on the findings of the present study, we hypothesize that plasmid encoded iron acquisition systems may be associated with biological functions distinct to that of chromosome encoded iron acquisition systems. Nonetheless, further study is necessary to identify the specific functions of plasmid-encoded factors including iron acquisition systems in the virulence of *Salmonella*. The present study further supports the necessity of understanding the roles of plasmid-encoded factors in virulence and pathogenesis of *Salmonella*.

## Additional files

Additional file 1: **Table S1.** List of genes that were upregulated in TC in ID as compared to IR growth conditions. (XLSX 4483 kb)

Additional file 2: **Table S2.** List of genes that were down regulated in TC in ID as compared to IR growth conditions. (XLSX 4349 kb)

Additional file 3: **Table S3.** List of genes that were upregulated in Wt in ID as compared to IR growth conditions. (XLSX 39 kb)

Additional file 4: **Table S4.** List of genes that were down regulated in Wt in ID as compared to IR growth conditions. (XLSX 39 kb)

Additional file 5: **Table S5.** Primers used in qRT-PCR for validation of RNA-Seq data. (DOCX 12 kb)

## Abbreviations

Coli-T: Colicin transporter gene
ID: Iron-deplete
IncFIB: Incompatibility group (Inc) FIB plasmid
IR: Iron-rich
qRT-PCR: Quantitative reverse transcription polymerase chain reaction
R: Recipient
*S. enterica*: *Salmonella enterica*
TC: Transconjugant
Wt: Wild type

## References

[1] Scallan E, Hoekstra RM, Angulo FJ, Tauxe RV, Widdowson MA, Roy SL, Jones JL, Griffin PM (2011). Foodborne illness acquired in the United States--major pathogens. Emerg Infect Dis.

[2] Scallan E, Mahon BE, Hoekstra RM, Griffin PM (2013). Estimates of illnesses, hospitalizations and deaths caused by major bacterial enteric pathogens in young children in the United States. Pediatr Infect Dis J.

[3] Gambino-Shirley K, Stevenson L, Concepcion-Acevedo J, Trees E, Wagner D, Whitlock L, Roberts J, Garrett N, Van Duyne S, McAllister G, et al. Flea market finds and global exports: four multistate outbreaks of human *Salmonella* infections linked to small turtles, United States-2015. Zoonoses Public Health. 2018. 10.1111/zph.12466 29577654

[4] Anderson TC, Marsden-Haug N, Morris JF, Culpepper W, Bessette N, Adams JK, Bidol S, Meyer S, Schmitz J, Erdman MM (2017). Multistate outbreak of human *Salmonella* typhimurium infections linked to pet hedgehogs - United States, 2011-2013. Zoonoses Public Health.

[5] Jones TF, Ingram LA, Cieslak PR, Vugia DJ, Tobin-D'Angelo M, Hurd S, Medus C, Cronquist A, Angulo FJ (2008). Salmonellosis outcomes differ substantially by serotype. J Infect Dis.

[6] Foley SL, Johnson TJ, Ricke SC, Nayak R, Danzeisen J (2013). *Salmonella* pathogenicity and host adaptation in chicken-associated serovars. Microbiol Mol Biol Rev.

[7] Uzzau S, Brown DJ, Wallis T, Rubino S, Leori G, Bernard S, Casadesus J, Platt DJ, Olsen JE (2000). Host adapted serotypes of *Salmonella enterica*. Epidemiol Infect.

[8] Galan JE (1996). Molecular genetic bases of *Salmonella* entry into host cells. Mol Microbiol.

[9] Winnen B, Schlumberger MC, Sturm A, Schupbach K, Siebenmann S, Jenny P, Hardt WD (2008). Hierarchical effector protein transport by the *Salmonella* typhimurium SPI-1 type III secretion system. PLoS One.

[10] Schlumberger MC, Hardt WD (2006). *Salmonella* type III secretion effectors: pulling the host cell's strings. Curr Opin Microbiol.

[11] Galan JE, Wolf-Watz H (2006). Protein delivery into eukaryotic cells by type III secretion machines. Nature.

[12] Gaviria-Cantin T, El Mouali Y, Le Guyon S, Romling U, Balsalobre C (2017). Gre factors-mediated control of *hilD* transcription is essential for the invasion of epithelial cells by *Salmonella enterica* serovar typhimurium. PLoS Pathog.

[13] Sabbagh SC, Forest CG, Lepage C, Leclerc JM, Daigle F (2010). So similar, yet so different: uncovering distinctive features in the genomes of *Salmonella enterica* serovars typhimurium and Typhi. FEMS Microbiol Lett.

[14] Hensel M, Shea JE, Waterman SR, Mundy R, Nikolaus T, Banks G, Vazquez-Torres A, Gleeson C, Fang FC, Holden DW (1998). Genes encoding putative effector proteins of the type III secretion system of *Salmonella* pathogenicity island 2 are required for bacterial virulence and proliferation in macrophages. Mol Microbiol.

[15] Collazo CM, Galan JE (1997). The invasion-associated type-III protein secretion system in *Salmonella*--a review. Gene.

[16] Deiwick J, Nikolaus T, Shea JE, Gleeson C, Holden DW, Hensel M (1998). Mutations in *Salmonella* pathogenicity island 2 (SPI2) genes affecting transcription of SPI1 genes and resistance to antimicrobial agents. J Bacteriol.

[17] Walthers D, Carroll RK, Navarre WW, Libby SJ, Fang FC, Kenney LJ (2007). The response regulator SsrB activates expression of diverse *Salmonella* pathogenicity island 2 promoters and counters silencing by the nucleoid-associated protein H-NS. Mol Microbiol.

[18] Khajanchi BK, Hasan NA, Choi SY, Han J, Zhao S, Colwell RR, Cerniglia CE, Foley SL (2017). Comparative genomic analysis and characterization of incompatibility group FIB plasmid encoded virulence factors of *Salmonella enterica* isolated from food sources. BMC Genomics.

[19] Gokulan K, Khare S, Rooney AW, Han J, Lynne AM, Foley SL (2013). Impact of plasmids, including those encodingVirB4/D4 type IV secretion systems, on *Salmonella enterica* serovar Heidelberg virulence in macrophages and epithelial cells. PLoS One.

[20] Guiney DG, Fierer J (2011). The role of the *spv* genes in *Salmonella* pathogenesis. Front Microbiol.

[21] Schaible UE, Kaufmann SH (2004). Iron and microbial infection. Nat Rev Microbiol.

[22] Litwin CM, Calderwood SB (1993). Role of iron in regulation of virulence genes. Clin Microbiol Rev.

[23] Porcheron G, Dozois CM (2015). Interplay between iron homeostasis and virulence: Fur and RyhB as major regulators of bacterial pathogenicity. Vet Microbiol.

[24] Mey AR, Wyckoff EE, Kanukurthy V, Fisher CR, Payne SM (2005). Iron and fur regulation in *Vibrio cholerae* and the role of fur in virulence. Infect Immun.

[25] Ernst FD, Bereswill S, Waidner B, Stoof J, Mader U, Kusters JG, Kuipers EJ, Kist M, van Vliet AH, Homuth G (2005). Transcriptional profiling of *Helicobacter pylori* Fur- and iron-regulated gene expression. Microbiology.

[26] Troxell B, Sikes ML, Fink RC, Vazquez-Torres A, Jones-Carson J, Hassan HM (2011). Fur negatively regulates *hns* and is required for the expression of HilA and virulence in *Salmonella enterica* serovar typhimurium. J Bacteriol.

[27] Harrison A, Santana EA, Szelestey BR, Newsom DE, White P, Mason KM (2013). Ferric uptake regulator and its role in the pathogenesis of nontypeable *Haemophilus influenzae*. Infect Immun.

[28] Pich OQ, Merrell DS (2013). The ferric uptake regulator of *Helicobacter pylori*: a critical player in the battle for iron and colonization of the stomach. Future Microbiol.

[29] Torres VJ, Attia AS, Mason WJ, Hood MI, Corbin BD, Beasley FC, Anderson KL, Stauff DL, McDonald WH, Zimmerman LJ (2010). *Staphylococcus aureus* fur regulates the expression of virulence factors that contribute to the pathogenesis of pneumonia. Infect Immun.

[30] Ratledge C, Dover LG (2000). Iron metabolism in pathogenic bacteria. Annu Rev Microbiol.

[31] Kaplan J (2002). Mechanisms of cellular iron acquisition: another iron in the fire. Cell.

[32] Hentze MW, Muckenthaler MU, Andrews NC (2004). Balancing acts: molecular control of mammalian iron metabolism. Cell.

[33] Di Lorenzo M, Stork M. Plasmid-encoded Iron uptake systems. Microbiol Spectr. 2014;2(6). 10.1128/microbiolspec.PLAS-0030-2014 26104436

[34] Janakiraman A, Slauch JM (2000). The putative iron transport system SitABCD encoded on SPI1 is required for full virulence of *Salmonella typhimurium*. Mol Microbiol.

[35] Han J, Gokulan K, Barnette D, Khare S, Rooney AW, Deck J, Nayak R, Stefanova R, Hart ME, Foley SL (2013). Evaluation of virulence and antimicrobial resistance in *Salmonella enterica* serovar Enteritidis isolates from humans and chicken- and egg-associated sources. Foodborne Pathog Dis.

[36] Srikumar S, Kroger C, Hebrard M, Colgan A, Owen SV, Sivasankaran SK, Cameron AD, Hokamp K, Hinton JC (2015). RNA-seq brings new insights to the intra-macrophage transcriptome of *Salmonella* typhimurium. PLoS Pathog.

[37] Fabrega A, Vila J (2013). *Salmonella enterica* serovar typhimurium skills to succeed in the host: virulence and regulation. Clin Microbiol Rev.

[38] Kroger C, Colgan A, Srikumar S, Handler K, Sivasankaran SK, Hammarlof DL, Canals R, Grissom JE, Conway T, Hokamp K (2013). An infection-relevant transcriptomic compendium for *Salmonella enterica* Serovar Typhimurium. Cell Host Microbe.

[39] Hautefort I, Thompson A, Eriksson-Ygberg S, Parker ML, Lucchini S, Danino V, Bongaerts RJ, Ahmad N, Rhen M, Hinton JC (2008). During infection of epithelial cells *Salmonella enterica* serovar typhimurium undergoes a time-dependent transcriptional adaptation that results in simultaneous expression of three type 3 secretion systems. Cell Microbiol.

[40] Khajanchi BK, Han J, Gokulan K, Zhao S, Gies A, Foley SL. Draft genome sequences of four *Salmonella enterica* strains isolated from Turkey-associated sources. Genome Announc. 2016;4(5). 10.1128/genomeA.01122-16 PMC5064110 27738037

[41] Bjarnason J, Southward CM, Surette MG (2003). Genomic profiling of iron-responsive genes in *Salmonella enterica* serovar typhimurium by high-throughput screening of a random promoter library. J Bacteriol.

[42] Zaini PA, Fogaca AC, Lupo FG, Nakaya HI, Vencio RZ, da Silva AM (2008). The iron stimulon of *Xylella fastidiosa* includes genes for type IV pilus and colicin V-like bacteriocins. J Bacteriol.

[43] Robinson MD, Smyth GK (2008). Small-sample estimation of negative binomial dispersion, with applications to SAGE data. Biostatistics.

[44] Robinson MD, McCarthy DJ, Smyth GK (2010). edgeR: a Bioconductor package for differential expression analysis of digital gene expression data. Bioinformatics.

[45] Kim SJ, Kweon O, Sutherland JB, Kim HL, Jones RC, Burback BL, Graves SW, Psurny E, Cerniglia CE (2015). Dynamic response of *Mycobacterium vanbaalenii* PYR-1 to BP Deepwater horizon crude oil. Appl Environ Microbiol.

[46] Radtke AL, O'Riordan MX (2006). Intracellular innate resistance to bacterial pathogens. Cell Microbiol.

[47] Paradkar PN, De Domenico I, Durchfort N, Zohn I, Kaplan J, Ward DM (2008). Iron depletion limits intracellular bacterial growth in macrophages. Blood.

[48] Collins HL (2003). The role of iron in infections with intracellular bacteria. Immunol Lett.

[49] Chlosta S, Fishman DS, Harrington L, Johnson EE, Knutson MD, Wessling-Resnick M, Cherayil BJ (2006). The iron efflux protein ferroportin regulates the intracellular growth of *Salmonella enterica*. Infect Immun.

[50] Miller JF, Mekalanos JJ, Falkow S (1989). Coordinate regulation and sensory transduction in the control of bacterial virulence. Science.

[51] Skaar EP (2010). The battle for iron between bacterial pathogens and their vertebrate hosts. PLoS Pathog.

[52] Fisher CR, Davies NM, Wyckoff EE, Feng Z, Oaks EV, Payne SM (2009). Genetics and virulence association of the *Shigella flexneri* sit iron transport system. Infect Immun.

[53] Runyen-Janecky LJ, Reeves SA, Gonzales EG, Payne SM (2003). Contribution of the *Shigella flexneri* sit, Iuc, and Feo iron acquisition systems to iron acquisition in vitro and in cultured cells. Infect Immun.

[54] Johnson TJ, Thorsness JL, Anderson CP, Lynne AM, Foley SL, Han J, Fricke WF, McDermott PF, White DG, Khatri M (2010). Horizontal gene transfer of a ColV plasmid has resulted in a dominant avian clonal type of *Salmonella enterica* serovar Kentucky. PLoS One.

[55] Metcalf D, Sharif S, Weese JS (2010). Evaluation of candidate reference genes in *Clostridium difficile* for gene expression normalization. Anaerobe.

[56] Botteldoorn N, Van Coillie E, Grijspeerdt K, Werbrouck H, Haesebrouck F, Donne E, D'Haese E, Heyndrickx M, Pasmans F, Herman L (2006). Real-time reverse transcription PCR for the quantification of the mntH expression of *Salmonella enterica* as a function of growth phase and phagosome-like conditions. J Microbiol Methods.

[57] Shah DH (2014). RNA sequencing reveals differences between the global transcriptomes of *Salmonella enterica* serovar enteritidis strains with high and low pathogenicities. Appl Environ Microbiol.

[58] Abrahams GL, Hensel M (2006). Manipulating cellular transport and immune responses: dynamic interactions between intracellular *Salmonella enterica* and its host cells. Cell Microbiol.

[59] Holden DW (2002). Trafficking of the *Salmonella* vacuole in macrophages. Traffic.

[60] Choi E, Kim H, Lee H, Nam D, Choi J, Shin D (2014). The iron-sensing fur regulator controls expression timing and levels of *Salmonella* pathogenicity island 2 genes in the course of environmental acidification. Infect Immun.

[61] Zaharik ML, Vallance BA, Puente JL, Gros P, Finlay BB (2002). Host-pathogen interactions: host resistance factor Nramp1 up-regulates the expression of *Salmonella* pathogenicity island-2 virulence genes. Proc Natl Acad Sci U S A.

[62] Khajanchi BK, Odeh E, Gao L, Jacobs MB, Philipp MT, Lin T, Norris SJ (2015). Phosphoenolpyruvate phosphotransferase system components modulate gene transcription and virulence of *Borrelia burgdorferi*. Infect Immun.

[63] Richard AL, Withey JH, Beyhan S, Yildiz F, DiRita VJ (2010). The *Vibrio cholerae* virulence regulatory cascade controls glucose uptake through activation of TarA, a small regulatory RNA. Mol Microbiol.

[64] Lloyd CR, Park S, Fei J, Vanderpool CK. The small protein SgrT controls transport activity of the glucose-specific phosphotransferase system. J Bacteriol. 2017;199(11). 10.1128/JB.00869-16 PMC5424253 28289085

[65] Ghosh S, Dureja C, Khatri I, Subramanian S, Raychaudhuri S, Ghosh S. Identification of novel small RNAs in *Burkholderia cenocepacia* KC-01 expressed under iron limitation and oxidative stress conditions. Microbiology. 2017. 10.1099/mic.0.000566 29099689

[66] Ren J, Sang Y, Qin R, Cui Z, Yao YF (2017). 6S RNA is involved in acid resistance and invasion of epithelial cells in *Salmonella enterica* serovar typhimurium. Future Microbiol.

[67] Spriewald S, Glaser J, Beutler M, Koeppel MB, Stecher B (2015). Reporters for single-cell analysis of Colicin Ib expression in *Salmonella enterica* Serovar typhimurium. PLoS One.

[68] Kaldhone PR, Han J, Deck J, Khajanchi B, Nayak R, Foley SL, Ricke SC (2018). Evaluation of the genetics and functionality of plasmids in incompatibility group I1-positive *Salmonella enterica*. Foodborne Pathog Dis.

[69] Fredericq P (1957). Colicins. Annu Rev Microbiol.

[70] Simmons KW, Wooley RE, Brown J (1988). Comparison of virulence factors and R plasmids of *Salmonella* spp. isolated from healthy and ill swine. Appl Environ Microbiol.

