# Supplementary material for: Global transcriptomic analyses of Salmonella enterica in Iron-depleted and Iron-rich growth conditions
Source: BMC Genomics. 2019 Jun 13;20:490. doi: 10.1186/s12864-019-5768-0 (PMC6567447; doi:10.1186/s12864-019-5768-0)
Supplement: Supplementary file 5 — Table S5. Primers used in qRT-PCR for validation of RNA-Seq data. (DOCX 12 kb) [file 12864_2019_5768_MOESM5_ESM.docx]

**Table S5.** Primers used in qRT-PCR for validation of RNA-Seq data

| Gene ID | Gene | Forward primer | Reverse primer |
| --- | --- | --- | --- |
| AY603_05415 | enterobactin transporter (*entS*) | GTCTGGATCGCTGGTGTTAT | GGTTTGCAGCAGAGTGTATTG |
| AY603_21220 | iron ABC transporter (*fepC*) | CCTTGAGCAATTGGGCATAAC | AAGTTCAGGGCGACGAATC |
| AY603_24045 | colicin transporter (Colicin-T) | GGATGCATTATCAGTGCCATTAC | GCCAGAATTCTTTCCCTGTAAAC |
| AY603_24505 | enolase | TCCCTGAGTGAAACCTTTGATG | GCGCATTGCAACTGTCATATC |
|  | *gmk* | TTGGCAGGGAGGCGTTT | GCGCGAAGTGCCGTAGTAAT |
|  | *adk* | CGCAAACGTCTGGTGGAATA | CCTGCGTACCGTCAACTTTAG |
